# Exploring the Non-Covalent Bonding in Water Clusters

**DOI:** 10.3390/ijms24065271

**Published:** 2023-03-09

**Authors:** Luis E. Seijas, Cesar H. Zambrano, Rafael Almeida, Jorge Alí-Torres, Luis Rincón, Fernando Javier Torres

**Affiliations:** 1 Grupo de Química Computacional y Teórica (QCT-UR), Escuela de Ingeniería Ciencia y Tecnología (EICT), Universidad del Rosario, Bogotá 111221, Colombia; 2 Grupo de Química Computacional y Teórica (QCT-USFQ), Departamento de Ingeniería Química, Universidad San Francisco de Quito, Diego de Robles y Vía Interoceánica, Quito 17-1200-841, Ecuador; 3 Laboratorio de Procesos Dinámicos en Química, Departamento de Química, Facultad de Ciencias, Universidad de Los Andes, Mérida 5101, Venezuela; 4 Departamento de Química, Universidad Nacional de Colombia, Av. Cra. 30 #45-03, Bogotá 111321, Colombia

**Keywords:** non-covalent interactions, hydrogen bonds, QTAIM, source function

## Abstract

QTAIM and source function analysis were used to explore the non-covalent bonding in twelve different water clusters (H_2_O)*_n_* obtained by considering *n* = 2–7 and various geometrical arrangements. A total of seventy-seven O−H⋯O hydrogen bonds (HBs) were identified in the systems under consideration, and the examination of the electron density at the bond critical point (BCP) of these HBs revealed the existence of a great diversity of O−H⋯O interactions. Furthermore, the analysis of quantities, such as |V(r)|/G(r) and H(r), allowed a further description of the nature of analogous O−H⋯O interactions within each cluster. In the case of 2-D cyclic clusters, the HBs are nearly equivalent between them. However, significant differences among the O−H⋯O interactions were observed in 3-D clusters. The assessment of the source function (SF) confirmed these findings. Finally, the ability of SF to decompose the electron density (*ρ*) into atomic contributions allowed the evaluation of the localized or delocalized character of these contributions to *ρ* at the BCP associated to the different HBs, revealing that weak O−H⋯O interactions have a significant spread of the atomic contributions, whereas strong interactions have more localized atomic contributions. These observations suggest that the nature of the O−H⋯O hydrogen bond in water clusters is determined by the inductive effects originated by the different spatial arrangements of the water molecules in the studied clusters.

## 1. Introduction

One of the most important principles in chemistry and material science states that the physicochemical properties of the matter do not depend solely on the type of atoms composing it, but they are also the result of the main interactions between its constituents at a given geometry [1]. Within this context, it can be claimed that, as the interatomic bonds determine the properties of the molecules [2,3], the interaction between molecules (and other building blocks) defines the main characteristics of the substances [4,5,6,7,8]. The latter is particularly evident in condensed phases where it has been deduced that the intermolecular interactions obey an interplay between dispersive and electrostatic forces [9]. Therefore, different types of intermolecular interactions can be readily defined in terms of the dispersive or electrostatic degree of the involved forces. The latter, also referred to as non-covalent interactions, are significantly important since they play a fundamental role in several scenarios, such as: the conformation of molecular crystals [10,11], the pairing of amino acids and their protein folding [12,13], the supramolecular assembly [14], the DNA structural stabilization [15], and others.

Hydrogen bonds, referred to as HBs hereinafter, are a particular case of non-covalent interactions that have gained a great deal of attention due to their peculiar nature and their ubiquitous presence in many chemical and biochemical systems. On the one hand, the definition of the HB as a classical bond is still at the center of intense scientific debate [16], but on the other hand, HBs with strengths comparable to weak covalent bonds have been reported [17]. This contradictory behavior has been explained by considering that, besides the dispersive and electrostatic components governing HBs, these interactions are highly dependent on the inductive (i.e., non-local) character of the chemical environment in which they are formed [9]. It is important to point out that the latter behavior explains the electron delocalization existing between the HB donor and acceptor water molecules, which in turn results in the partial shared-closed shell character of HB interactions [18]. Due to the aforementioned, HB networks are known to possess a cooperative (i.e., non-additivity) behavior. In this regard, it must be indicated that a thorough comprehension of the latter is an essential requirement for the development of reliable models for solvents. However, its full description through experimental as well as theoretical means remains a very challenging task [19,20].

Water is a solvent, considered as essential to biological and chemical processes [15], where HBs have certainly a key role. Thus, exploration of the structure and binding properties of small water clusters is of significant importance since it provides insights on the properties and behavior associated to the different condensed phases of water. Liquid water, the most common form on earth, has several unique properties, such as a maximum density occurring at 4 °C, a relatively high vapor pressure, the expansion of its volume upon freezing, and its exceptionally high surface tension [21]. It is also well-known that long-range order does not exist in liquid water [21,22]. Thus, the understanding of its local structure, i.e., the HB networks in water clusters, is inextricably tied to properly describing its distinctive properties. In addition to liquid phase, multiple ice phases, which dwell in the complicated phase diagram of this system, are also derived from distinct local environments, resulting in a wide range of densities, lattice energies, and other thermodynamic properties [21,22,23,24].

As commented before, the study of the HB networks present in water clusters is essential to obtain a proper description of water as a solvent. In this vein, small water clusters, (H_2_O)*_n_* with *n* < 10, have been intensively studied at different levels of calculation [25,26,27]. These reports have suggested that, on a microscopic scale, the density heterogeneity in liquid water is directly related to its local structure [28,29,30,31]. Related to the latter, Chaplin proposed a water model capable of switching from lower to higher density forms without breaking HBs [30]. For this dynamic behavior to be achieved, the model was constructed from various HB arrangements formed by a combination of solely hexamer and pentamer substructures [28,30]. Thus, a major finding of these works is that a precise description of the non-covalent bonding in small water clusters is accurate enough to investigate the structural changes as well as the dynamical behavior of water in its liquid state.

Before closing the present section, it is important to point out that many of the interesting properties of water have their origin in the non-additivity nature of the HBs (see above). In this context, Koehler et al., conducted ab initio calculations on the ground state of the linear water dimer with Cs symmetry and the cyclic water tetramer with S4 symmetry. This work demonstrated that an energy gain of 29% results from the cooperativity effects as estimated by using parameters based on two-body non-neighbor interaction energy as well as three- and four-body contributions [32]. Later, Suhai demonstrated that HB networks in ice are also the result of highly cooperative behavior. Moreover, this study also found that the cohesive energy of ice is the consequence of a complex interplay between repulsive and attractive terms, which exhibits different long-term interactions [33]. Likewise, other researchers investigated the significance of all higher-order interaction energy components, particularly the three-body factor among the non-additive terms for water clusters ranging from the trimer to the hexamer [34,35]. From the latter, it was suggested that non-additivity is more significant in HB networks belonging to small water clusters, where donor-acceptor arrangements involve the largest number of water molecules. At this point, it is important to comment that studies, such as those mentioned above as well as works cited in Refs. [36,37,38], are focused on structural or energetic aspects of the water clusters, but a detailed description of the nature of the non-covalent bonding on water clusters is still missing.

In this work, the HB network of small water clusters, ranging from the dimer to the heptamer, with different geometrical arrangements will be described by means of the quantum theory of atoms in molecules, QTAIM, and further analysis performed by employing the source function, SF. From there, the nature of the different HBs, O−H⋯O, found in the studied clusters will also be highlighted using quantities related to the Laplacian of the electron density. Regarding the adopted methodology, it must be indicated that it has been demonstrated to produce an accurate description of the intermolecular interactions in ion-solvent systems [39].

## 2. Results and Discussion

### 2.1. Geometric and Energetic Features of Water Clusters

**Figure 1:**
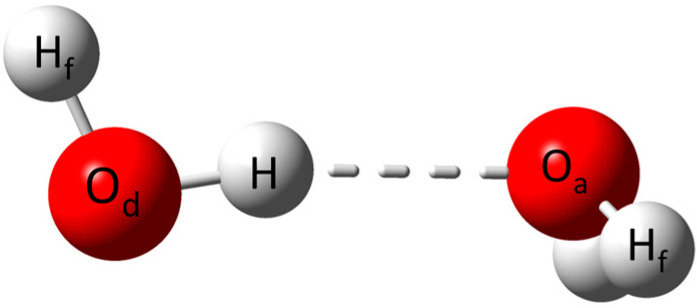
Water dimer describing the notation used to identify the atoms involved in the HBs.

The clusters adopted in the present study correspond to the models previously employed by Řezáč et al. [40], which have been recognized to provide an adequate representation of the water system. The atoms of each cluster involved in the HB interaction were denoted as O_d_–H⋯O_a_, referring to donor and acceptor oxygen atoms, as depicted in Figure 1. This notation will be upheld along the discussion. To understand the differences between the HBs formed in each cluster, the geometrical aspects of all the clusters are to be described. Moreover, for the sake of clearness, the models are divided into two subgroups: (i) 2-D clusters comprising the cyclic clusters (H_2_O)*_n_*, with *n* = 3–6 and (ii) 3-D clusters comprising the cage-like clusters (H_2_O)*_n_*, this being exclusively for *n* = 6–7.

**Figure 2:**
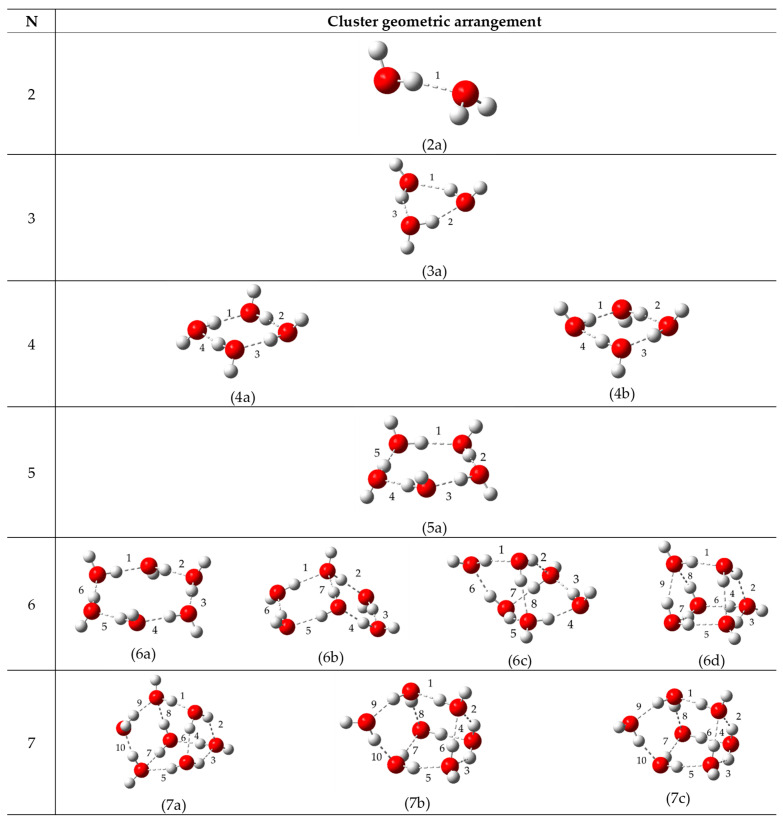
Equilibrium geometries of water clusters considered in the present study. Here 2a shows the dimer geometry, 3a, 4a, 4b, 5a and 6a shows the geometries of the 2D ring clusters. Finally, 6b, 6c, 6d, 7a, 7b and 7c depict the geometries for the 3D clusters.

Analysis of the equilibrium geometry of the models showed in Figure 2 (see Section 3) shows that the 2-D clusters are characterized by the shortening of the average O_d_⋯O_a_ distance as the cluster size increases. As reported in Table S1 (see Supplementary Materials), the O_d_⋯O_a_ interatomic distance changes from 2.78 Å to 2.70 Å for (H_2_O)_2_ and (H_2_O)_6_, respectively. Conversely, the donor covalent O_d_–H bond distance increases with the ring size, going from 0.87 Å to 0.98 Å for (H_2_O)_2_ and (H_2_O)_6_, respectively. Previous reports [34,35,41,42] have attributed this behavior to the non-additive, many-body nature of the systems. However, this trend is no longer observed in 3-D clusters. In those cases, the molecular arrangement allows the formation of a larger number of HBs with respect to the number of water molecules of the system. For instance, in the 2-D cluster 6a, the water molecules donate only one hydrogen atom, whereas in the 3-D cluster 6d three water molecules donate their two hydrogen atoms, increasing by three the number of total HBs (see Table 1). It is worth of mentioning that the additional HBs adopt a linear arrangement, which, as reported by Gosh et al. [43] for the water dimer, represents the most favorable geometry. However, the additionally formed HBs have, on average, longer distances than the ones observed in cyclic systems. For instance, in the case of cluster 6b, the HB O_d_⋯O_a_ distance is larger by 0.04 Å than in 6a. Despite this, three HBs are shorter than the average O_d_⋯O_a_ distance of 6a. Moreover, in 6b, the donor covalent O_d_–H bond distance is, on average, larger by 0.06 Å than 6a. Similar observations are made on cage-like clusters 6c and 6d, where the O_d_⋯O_a_ distances are, on average, 0.08 Å and 0.10 Å larger than those observed in 6a, whereas the donor covalent O_d_–H bond distances are larger by 0.12 Å and 0.18 Å. Finally, clusters 7a–c, corresponding to 3-D cage-like structures (Figure 2), are characterized by O_d_⋯O_a_ distances larger than those in 6a but shorter than those in 6d. These observations point out that clusters 6b–6d and 7a–7c have a greater diversity of O_d_–H⋯O_a_ HBs. It is important to mention that the O⋯O distances observed in clusters (H_2_O)_6_ and (H_2_O)_7_ are between the O_d_⋯O_a_ distances reported for the liquid water [44] and hexagonal Ice I_h_ [45].

Regarding the energetic aspects, Table 1 shows the calculated binding energies per water molecule for the twelve fully optimized clusters. The binding energies, $BE\left[{\left(H_{2}O\right)}_{n}\right]$, have been calculated as the negative of the difference between the total counterpoise (CP) corrected energy of the cluster, $E\left[{\left(H_{2}O\right)}_{n}\right]$, and the sum of the energies of the optimized monomers, $E\left[H_{2}O\right]$,

(1) $$BE\left[{\left(H_{2}O\right)}_{n}\right]=-\left(E\left[{\left(H_{2}O\right)}_{n}\right]-nE\left[H_{2}O\right]\right).$$

From Table 1, it can be noticed that our counterpoised corrected binding energies computed at the M062X/aug-cc-pVTZ level are comparable with the values obtained at CCSD(T)/CBS/CBS level reported in Ref. [46], a fact that validates the method employed in the present study. It is also observed in Table 1 that the BE per water molecule increases with respect to the number of molecules of the system, as expected. Although the latter definition of BE is customarily used to describe the energetic features of molecular aggregates, in the case of water clusters, it is also interesting to consider the BE per HB, because it provides an average of the stabilization energy gained per HB formed in each system. As reported in Table S2 (see Supplementary Materials), the BE per HB systematically increases with the system size for the case of 2-D cyclic clusters. However, when the more complex 3-D structures are considered, this value decreases as the number of HBs increases. The latter is particularly clear in the hexamers, where, given a fixed number of molecules (i.e., *n* = 6), the HBs go from 6 to 9. The data summarized in Table S2 shows that the BE per HB is 7.90 in the 2-D cyclic cluster 6a, whereas this quantity decreases to 5.71 in the 3-D cluster 6d. The last result allows us to conclude that the more complex the geometrical arrangement of the cluster, the lower the average contribution of each HB to the total energy of the system, suggesting that, while in 2-D structures the HBs formed are equivalent, in the 3-D systems a larger diversity of HBs can be expected.

**Figure 3:**
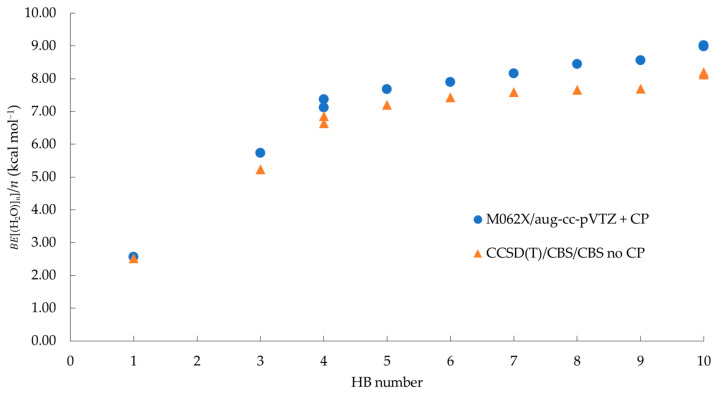
Binding energies per water molecule $BE\left[{\left(H_{2}O\right)}_{n}\right]/n$ in kcal mol^−1^ vs. the number of HB formed.

Despite the significant differences between the BE per water molecule and its counterpart with respect to HBs formed, the plot depicted in Figure 3 shows that $BE\left[{\left(H_{2}O\right)}_{n}\right]$/*n* increases with the total number of HB formed. Thus, these results indicate that the $BE\left[{\left(H_{2}O\right)}_{n}\right]$ depends on two main factors: (i) the cluster size and (ii) the number of formed HBs, a behavior that has been also attributed to the non-additive nature of the HB network (Figure 3) [34,35,41,42]. The data summarized in Table 1 show that, in the case of the 2-D tetramers (H_2_O)_4_, a difference of 0.26 kcal mol^−1^ is observed between 4a and 4b, this being attributed to the relative orientation of free hydrogen atoms: in 4a, the free hydrogen atoms of neighboring molecules are oriented at the same side of the main plane (Figure 2c), whereas the hydrogen atoms show an alternate orientation in 4b (Figure 2d). On the other hand, clusters 6a, 6b, and 6c have slightly lower binding energies than 6d (0.26 kcal mol^−1^, 0.40 kcal mol^−1^ and 0.03 kcal mol^−1^, respectively). Finally, the 3-D clusters 7a, 7b, and 7c, can be considered as quasi-degenerated structures. In the next sections, the non-covalent bonding in all the considered water clusters will be analyzed.

### 2.2. QTAIM

As the second stage of this work, the HB interactions in each cluster were analyzed using the quantum theory of atoms in molecules (QTAIM) [47]. This theory represents a powerful tool for describing different types of chemical interactions, including non-covalent interactions. Some examples of this affirmation can be found in Refs. [48,49,50,51]. Within the QTAIM, a zero-flux surface defines each atom’s boundary in the electron density gradient vector field. This partition allows the separation of regions, Ω, identified as atoms in molecules. Moreover, the topological analysis of the electron density allows the identification of a set of critical points that occurs when the gradient vanishes (i.e., ∇ρ=0). This set of points can be classified using another quantity derived from the electron density, that is the Laplacian of the density $\nabla^{2}\rho$. This quantity can be written as the sum of contributions along the three principal axes of maximum variation, namely the eigenvalues ($\lambda_{i}$) of the Hessian matrix: $\nabla^{2}\rho =\lambda_{1}+\lambda_{2}+\lambda_{3}$. Furthermore, the algebraic sum of the signs of $\lambda_{i}$, usually referred to as the signature (σ), together with the number of the non-zero curvatures, also known as rank (*ω*), allows the classification of the electron density critical points. Four different types of critical points can be found: (i) local maxima (3, −3), associated to nuclear critical points; (ii) saddle points (3, −1), associated to bond critical points; (iii) saddle points (3, +1), associated to ring critical points; and (iv) local minima (3, +3), associated to cage critical points. In this work, the HBs will be characterized by evaluating different quantities derived from the electron density at the bond critical points (BCP). The importance of the latter ones can be understood if two important aspects are considered: (i) at the BCP, $\rho \left(r_{BCP}\right)$ has the smallest value of the electron density along the bond path, and (ii) it corresponds to the maximum of density at the interatomic surface between the two atoms. Therefore, these two important properties of $\rho \left(r_{BCP}\right)$ will be used to characterize the HBs in the considered water clusters.

**Figure 4:**
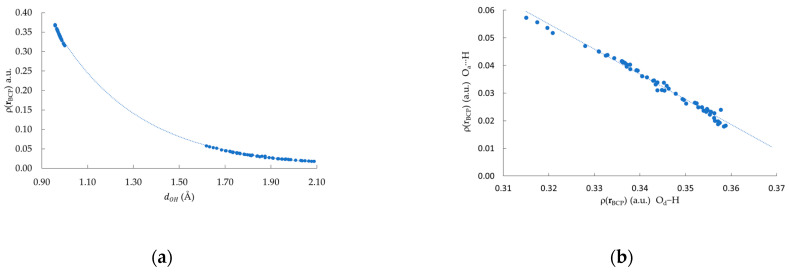
(**a**) Relationship between $d_{OH}$ distance (Å) and $\rho \left(r_{BCP}\right)$ (a.u.). The fitted curve corresponds to $\rho \left(r_{BCP}\right)=5.380e^{-2.810d_{OH}}$, $r^{2}=0.9996$, $d_{OH}$ makes reference to the distances between either O_d_ H or O_a_ H; (**b**) Relationship between $\rho \left(r_{BCP}\right)$ at Od–H and $\rho \left(r_{BCP}\right)$ at O_a_⋯H.

In all the clusters, two different BCPs can be found, one corresponding to the covalent O_d_–H polar bonds and the second related to the non-covalent HB interaction O_a_⋯H, following the notation depicted in Figure 1. As we mention before, the uniqueness of the BCP allows the characterization of the whole bond in terms of different properties of $\rho \left(r_{BCP}\right)$. Figure 4a depicts the $\rho \left(r_{BCP}\right)$ dependence with the two oxygen-hydrogen interatomic distances; namely: O_d_–H and O_a_⋯H. From Figure 4, two sets of points are clearly identified, one at shorter distances, associated with the O_d_–H bonds, and the other one, at larger distances, corresponding to the O_a_⋯H HBs. Moreover, these $\rho \left(r_{BCP}\right)$ vs. $d_{OH}$ points fit an exponential function [$\rho \left(r_{BCP}\right)=5.380e^{-2.810d_{OH}}$, $r^{2}=0.9996$]. This result shows that both types of bond O_d_–H and O_a_⋯H fit an empirical model previously reported for other systems [52,53]. From Figure 4b, an inverse relationship between the $\rho \left(r_{BCP}\right)$ value computed at the O_d_–H BCP and its O_a_⋯H counterpart is observed. This last result suggests that a charge transfer occurs from the covalent O_d_–H bond to the O_a_⋯H HB. It is important to comment that this has also been observed in other systems characterized by N−H⋯N and F−H⋯F HB interactions [18,54].

The previously discussed results show that a wide variety of HBs are present in the studied models, being characterized by different O_a_⋯H distances and $\rho \left(r_{BCP}\right)$ values computed at the BCP. Therefore, to obtain a more complete description of the HB network, other topological properties are needed. One quantity that can be employed for this purpose is the Laplacian of the density, $\nabla^{2}\rho$, because it not only provides a classification of the BCPs, but its sign can be used to distinguish between regions of concentration or depletion of the electron density with respect to the surroundings. In fact, for pure shared-shell interactions, $\nabla^{2}\rho <0$, whereas, for pure closed-shell interactions, $\nabla^{2}\rho >0$. However, in the transit region from closed-shell to shared-shell, $\nabla^{2}\rho$ presents a particular behavior, first increasing until reach a maximum and then decreasing [55]. The latter makes $\nabla^{2}\rho$ alone not fully adequate to study HB interactions that indeed belong to the aforementioned transit region. Thus, a more convenient quantity to characterize the O_a_⋯H HBs would be the total electron energy density, H(r), defined as follows:

(2) H(r)=G(r) + V(r),

where G(r) and V(r) are the local kinetic and potential density energies. It is worth of mentioning that H(r) is connected to the Laplacian through the local virial theorem:

(3) $$\frac{1}{4}\nabla^{2}\rho \left(r\right)=2G\left(r\right)\;+\;V\left(r\right).$$

Because V(r) is always negative and G(r) is always positive, a local concentration of the electron density implies that the local potential energy density exceeds twice the kinetic energy density, whereas a local depletion of the electron energy corresponds to the opposite situation. The use of the local virial theorem in the H(r) definition (Equation (2)), results in the following expression:

(4) $$H\left(r\right)=\left(1/2\right)\left[V\left(r\right)+\left(1/4\right)\nabla^{2}\rho \left(r\right)\right],$$

where the second term measures the concentration or depletion of the charge in the interatomic region. From Equation (4), it can be deduced that the more dominant the character of V(r) the more negative are the values of H(r), which results in a further stabilization of the molecular system. In this context, analysis of the local properties of $V\left(r_{BCP}\right)$ and $G\left(r_{BCP}\right)$ are adequate for the description of chemical bonding and intermolecular interactions [56,57]. From all above, it can be deduced that $H\left(r_{BCP}\right)<0$, and $\nabla^{2}\rho \left(r_{BCP}\right)>0$ are conditions associated to the transit region from closed-shell to share-shell interactions; whereas, $H\left(r_{BCP}\right)>0$, and $\nabla^{2}\rho \left(r_{BCP}\right)>0$ are conditions associated to pure closed-shell interactions. On the other hand, the value of the $\left|V\left(r_{BCP}\right)\right|/G\left(r_{BCP}\right)$ quotient can be divided in three types: (i) $\left|V\left(r_{BCP}\right)\right|/G\left(r_{BCP}\right)>2$, associated to shared-shell interactions; (ii) $1<\left|V\left(r_{BCP}\right)\right|/G\left(r_{BCP}\right)<2$, associated to the transit region from closed-shell to share-shell interaction; and (iii) $\left|V\left(r_{BCP}\right)\right|/G\left(r_{BCP}\right)<1$ associated to pure closed-shell interactions. It is important to point out that the described method has been successfully employed to classify HBs in biuret–water systems [58].

**Figure 5:**
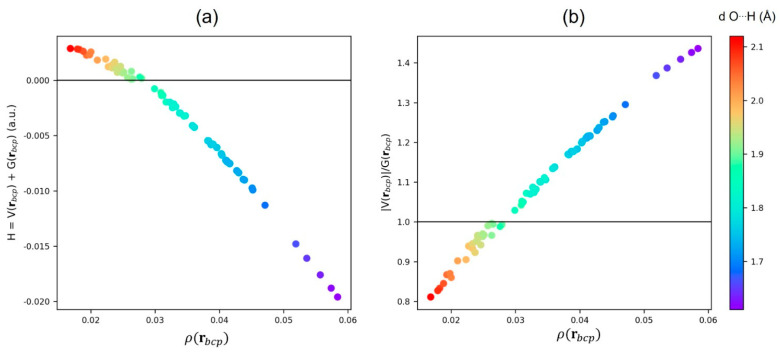
(**a**) Relationship between $H\left(r_{BCP}\right)$ (a.u.) with the HB $\rho \left(r_{BCP}\right)$ (a.u) and (**b**) Relationship between $\left|V\left(r_{BCP}\right)\right|/G\left(r_{BCP}\right)$ (a.u.) with the HB $\rho \left(r_{BCP}\right)$ (a.u). The color scale it related to the interatomic distance O_a_⋯H.

As reported in Table S1 (see Supplementary Materials), HBs in water dimer (2a) and trimer (3a) are characterized by $H\left(r_{BCP}\right)>0$ (0.0007 a.u. and 0.0008 a.u., respectively) and $\left|V\left(r_{BCP}\right)\right|/G\left(r_{BCP}\right)<1$ (0.966 and 0.988, respectively). On the contrary, in the 4a, 4b, 5a, and 6a clusters, $H\left(r_{BCP}\right)<0$ and $1<\left|V\left(r_{BCP}\right)\right|/G\left(r_{BCP}\right)<2$. In these clusters, a change in the value of the ratio $\left|V\left(r_{BCP}\right)\right|/G\left(r_{BCP}\right)$ suggests a strong reorganization of $\rho \left(r_{BCP}\right)$ associated to the shortening of the O_a_⋯H distance. Nevertheless, in three dimensional hexamers, 6b, 6c, and 6d, $H\left(r_{BCP}\right)$ shows both positive and negative values, being the extreme cases those of the 6d cluster where $H\left(r_{BCP}\right)$ varies form −0.0161 a.u. to 0.0028 a.u. In agreement, the $\left|V\left(r_{BCP}\right)\right|/G\left(r_{BCP}\right)$ ratio takes values between 0.833 and 1.387. These observations indicate that the HBs in 3-D clusters have different nature as compared to its 2-D cyclic counterparts, and they are highly dependent on the electronic and geometric features of the systems. As the O_a_⋯H distance shortens and the O_a_⋯H—O_d_ angle becomes more linear (see Table S1), $H\left(r_{BCP}\right)$ takes more negative values, whereas the $\left|V\left(r_{BCP}\right)\right|/G\left(r_{BCP}\right)$ ratio increases, suggesting that the interaction becomes stronger as observed by the increase of $\rho \left(r_{BCP}\right)$ (see Figure 5). Moreover, despite the $\nabla^{2}\rho \left(r_{BCP}\right)>0$ for all the O_a_⋯H interactions, it can be noticed that the larger the magnitude of $\left|V\left(r_{BCP}\right)\right|$ with respect to $G\left(r_{BCP}\right),$ the lower the $\nabla^{2}\rho \left(r_{BCP}\right)$ value.

**Figure 6:**
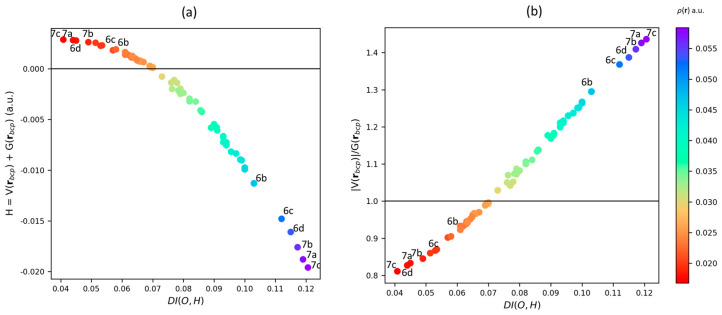
(**a**) Relationship between $H\left(r_{BCP}\right)$ (a.u.) with the H⋯O_a_ DI (a.u.) and (**b**) Relationship between $\left|V\left(r_{BCP}\right)\right|/G\left(r_{BCP}\right)$ (a.u.) with the H⋯O_a_ DI. The color scale it related to the HB $\rho \left(r_{BCP}\right)$ (a.u.).

Additional insights on the nature of the HBs in the different water clusters can be gained by employing the delocalization index, DI, defined as follows:

(5) $$\mathrm{DI}\left(\Omega_{A},\Omega_{B}\right)=-2\int_{\Omega_{A},\Omega_{B}}^{}\left(2\Gamma \left(r_{1},\;r_{2}\right)-\rho \left(r_{1}\right)\rho \left(r_{2}\right)\right)dr_{1}dr_{2},$$

where ρ(r) and $\Gamma \left(r_{1},\;r_{2}\right)$ correspond to the one and pair densities, respectively. As its name states, the DI quantity is related to the number of electrons delocalized between atoms A and B. This quantity can be interpreted as the fraction of electrons that are shared in the classical Lewis model, and it has been successfully used as a descriptor to analyze HBs [59,60]. In this context, the DI is associated with the shared-shell character of an intermolecular bond [60,61]. DI(O_a_,H) computed for the different HBs in the studied water clusters ranges from 0.041 to 0.121. In the particular case of 2-D clusters, the DI(O_a_,H) values obtained for the HBs belonging to a given cluster are very close (see Table S3). However, as the number of water molecules grows, the average DI value also increases going from 0.066 for the dimer 2a to 0.094 for the hexamer 6a. On the other hand, as the O_a_⋯H interatomic distance takes different values in 3-D clusters, the DI(O_a_,H) also spans a wide range of values. For instance, in the cluster 6d, the strongest O_a_⋯H has a DI(O_a_,H) value of 0.115, being 2.5 times the DI(O_a_,H) observed for the weakest O_a_⋯H. Moreover, clusters, 7a, 7b, and 7c possess the highest DI(O_a_,H) values, 0.199, 0.117, and 0.121, respectively, but these clusters also have the lowest DI(O_a_,H) values, being 0.044, 0.051, and 0.041 for 7a, 7b, and 7c, respectively. Figure 6 shows the plots of the bond descriptors $H\left(r_{BCP}\right)$ and $\left|V\left(r_{BCP}\right)\right|/G\left(r_{BCP}\right)$ versus the DI(O_a_,H) for the intermolecular O_a_⋯H interactions. From Figure 6, a clear relationship between the DI(O_a_,H) and the two descriptors is observed, where the shared-shell character of the interaction grows as the DI(O_a_,H) values increases. The latter shows that different types of O_a_⋯H interactions are present in water clusters as suggested before. It is worth noting that this behavior also supports the idea that HBs in water clusters do not obey a pairwise additive rule. Indeed, at this stage, it is possible to establish that the results presented above indicate that each individual water molecule affects the entire HB network. Moreover, the influence of each water molecule can vary significantly in 3-D clusters, suggesting that an accurate enough molecular potential must consider the inductive (i.e., non-local) effect of each molecule of the system over the whole HBs network. To further explore the latter conjectures, the non-local effects of the water clusters on their HB network will be analyzed by means of the source function in the following section.

### 2.3. Source Function Analysis

The non-local effects that stabilize the O_a_⋯H interactions present in the considered water clusters were analyzed by adopting the source function (SF) introduced by Bader and Gatti in 1998 [62]. This function can be applied to decompose the electron density at **r** as the sum of contributions of different local sources $LS\left(r,r^{\prime }\right)$, operating at all the other points of the space [63,64]. Within this context, the electron density can be written as:

(6) $$\rho \left(r\right)\;=\;\overset{\;}{\int }LS\left(r,r^{\prime }\right)dr^{\prime }$$

where the local source is given by $LS\left(r,r^{\prime }\right)=-{\left(4\pi \left|r-r^{\prime }\right|\right)}^{-1}\;\cdot \nabla^{2}\rho \left(r^{\prime }\right)$. As observed in its definition, *LS* represents how the Laplacian of the electron density at the position $r^{\prime }$ affects the electron density at a different position r [25]. Accordingly, ρ(r) can be written as a sum of atomic source contributions:

(7) $$\rho \left(r\right)\;=\;\sum_{\Omega }\;S\left(r,\;\Omega \prime \right),$$

where *S*, referred to as source function (SF), is defined as:

(8) $$S\left(r,\;\Omega \right)=\int_{\Omega }LS\left(r,r^{\prime }\right)\;\cdot dr^{\prime }.$$

Equation (7) implies that ρ(r) is given by the contribution, S(r, Ω), of each atomic basin of the complete system, indicating that SF provides a measure of the relative contribution of an atom or group of atoms to the electron density at r [47]. The physical meaning of this contributions can be understood employing the local expression of the virial theorem (Equation (3)) in the $LS\left(r,r^{\prime }\right)$ definition to obtain the following:

(9) $$LS\left(r,r^{\prime }\right)\;=-\frac{1}{\pi }\times \frac{2G\left(r^{\prime }\right)+V\left(r^{\prime }\right)}{\left|r-r^{\prime }\right|},$$

where $G\left(r^{\prime }\right)$ is the positively defined kinetic energy density, and $V\left(r^{\prime }\right)$ is the electronic potential energy. According to Equation (9), molecular regions, where the electron density is concentrated ($V\left(r^{\prime }\right)>2G\left(r^{\prime }\right)$), are sources for the electron density at r. On the other hand, regions where electron density is depleted ($V\left(r^{\prime }\right)<2G\left(r^{\prime }\right)$), acts as sinks for ρ(r). It is worth to mention that the capability of the electron density at $r^{\prime }$ to be a source (or a sink) at another point r is related to the magnitude of its charge concentration or depletion (magnitude of $\nabla^{2}\rho \left(r^{\prime }\right)$), weighted by the inverse of the distance between these two points [64]. Here, for the sake of simplicity, the source function values will be expressed as the percentage contribution to the electron density as:

(10) $$S\%\left(r,\Omega \right)=\left[\frac{S\left(r,\Omega \right)}{\rho \left(r\right)}\right]\times 100.\;$$

It is important to point out that S%(r,Ω) represents the percentage share of electron density from Ω to ρ(r). Thus, in principle, S%(r,Ω) reflects the delocalized or localized character of a chemical interaction. For two bonded atoms S%(r,Ω) will be large if the interaction is localized and will be small for highly delocalized interactions.

The SF contributions to the O_a_⋯H electronic density at the bond critical point, $\rho \left(r_{BCP}\right)$, were examined to investigate the nature of the HB in water clusters. The H atom acts as source for the BCP electron density in the O_d_—H covalent bond, with SF contributions ranging from 35.00% to 39.00%. Thus, the O_d_ atom contributions range from 59.93% to 61.00%. This finding reflects the shared-shell character of the O_a_⋯H interactions ($\nabla^{2}\rho \left(r_{BCP}\right)<0$), and the $S\%\left(O_{d},H\right)$ values, 96.92% on average, suggest that charge contributions are localized on the interacting atoms. For more information, see Table S4. On the other hand, the H atom acts as a sink on the O_a_⋯H interactions with S%(H) values starting at −48.82%. In contrast to the behavior observed on the O_d_–H covalent interactions, S%(H) values barely exceed zero in the strongest HB on (H_2_O)_7_ clusters (see Table S4). In 2-D clusters, S%(H) increases with the number of water molecules, suggesting that the positive contributions of $\nabla^{2}\rho \left(r^{\prime }\right)$ within the hydrogen basin decreases in comparison to negative contributions to O_a_⋯H $\rho \left(r_{BCP}\right)$. In other words, in the H basin, the charge concentration regions become more important than the charge depletion regions; thus, hydrogen atoms became a more significant source as the number of water molecules increases in the system. The average source contribution at $\rho \left(r_{BCP}\right)$ for the HB interactions in 2-D clusters are shown in Table 2. From this Table, it can be concluded that the larger the $\left|V\left(r_{BCP}\right)\right|/G\left(r_{BCP}\right)$ ratio, the larger S%(H) and $S\%\left(O_{a}\right)$, but the lower $S\%\left(O_{d}\right)$. The latter results also indicate that as the HB increase its shared-shell character, the $\rho \left(r_{BCP}\right)$ is mainly due to the O_d_ and O_a_ atomic contributions; because, as was mentioned before, the hydrogen atoms act as sinks. Furthermore, the increase in $S\%\left(O_{d},H,O_{a}\right)$ values can be attributed to a localization of the atomic density charge contributions.

**Figure 7:**
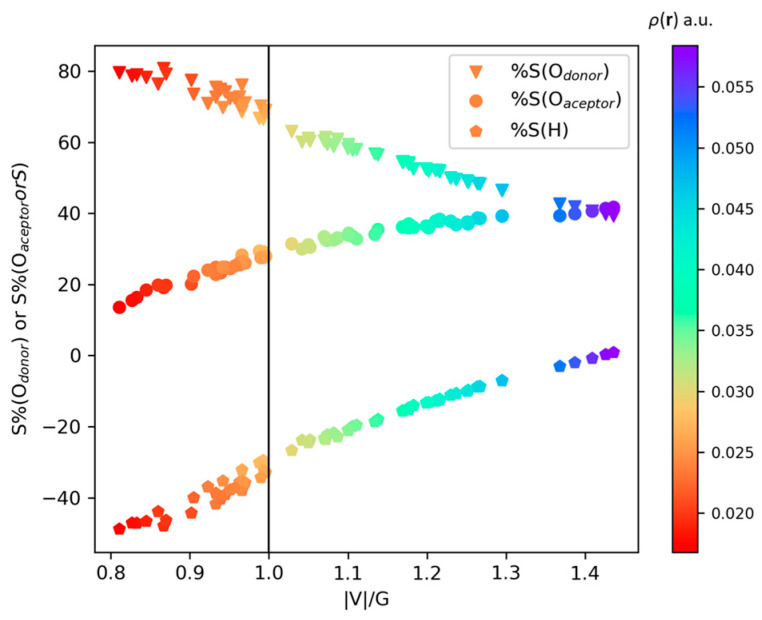
Relationship between atomic SF contributions to the HB $\rho \left(r_{BCP}\right)$ (a.u.) with the ratio |V(r)|/G(r) (a.u.). The color scale is related to the HB $\rho \left(r_{BCP}\right)$ (a.u).

Figure 7 shows the atomic source contribution for all the HB in the considered clusters plotted against the ratio $\left|V\left(r_{BCP}\right)\right|/G\left(r_{BCP}\right)$, where it can be seen that S%(H) and $S\%\left(O_{a}\right)$ increase with the $\left|V\left(r_{BCP}\right)\right|/G\left(r_{BCP}\right)$ value, whereas $S\%\left(O_{d}\right)$ decreases until it becomes roughly equal to $S\%\left(O_{a}\right)$. The $S\%\left(O_{d},H,O_{a}\right)$ values span the 44.3–66.17% range for pure closed-shell interactions region, i.e., |V(r)|/G(r)<1, with the remaining contributions coming from atoms not directly participating in the HB. On the other hand, in the transit region, i.e., 1≤|V(r)|/G(r)<2, the contributions of the O_d_−H⋯O_a_ triad ranges from 66.19% to 81.84%. It is worth noting that an increase in the percentage contribution of the triad O_d_−H⋯O_a_ can be directly related to an increase in the ratio $\left|V\left(r_{BCP}\right)\right|/G\left(r_{BCP}\right)$, which, in turn, can be associated to an increase in the interaction’s shared-shell nature.

**Figure 8:**
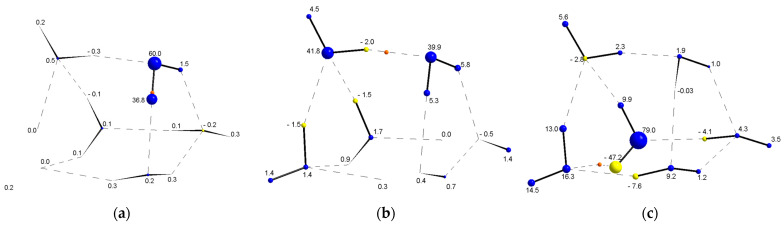
Pictorial representation of the SF contributions to $\rho \left(r_{BCP}\right)$ at (**a**) a O_d_–H covalent bond, (**b**) strong O_a_⋯H HB (i.e, $1<\left|V\left(r_{BCP}\right)\right|/G\left(r_{BCP}\right)<2$ ) and (**c**) weak O_a_⋯H HB (i.e., $\left|V\left(r_{BCP}\right)\right|/G\left(r_{BCP}\right)<1$ ). Blue color represents positive contributions (sources), whereas yellow color represents negative SF contributions (sinks).

The SF was employed also to unveil the character of the HBs in 3-D clusters. This analysis was focused on the 3-D cluster 6d as a representative case, because it contains HBs that belong to the pure closed-shell and the transit regions, as shown by the evaluation of $\left|V\left(r_{BCP}\right)\right|/G\left(r_{BCP}\right)$, $H\left(r_{BCP}\right)$, and DI(O,H) (see tables in Supplementary Material). In this case, the SF was employed to examine the localized/delocalized character of the atomic contributions to the $\rho \left(r_{BCP}\right)$ of the O_a_⋯H interaction. These atomic contributions are calculated by integrating the local source, $LS\left(r,r^{\prime }\right)$, on the atomic basin (see Equations (7)–(9). It is important to mention that, since the topography of the electron density at any location in space affects the electron density at the O_a_⋯H BCP, the atomic SF contribution can be used as an alternative to explore electron delocalization [64,65,66,67]. Figure 8 displays a pictorial description of the atomic source contributions to the electron density at the BCP of different bonds and interactions. For the O_d_–H covalent bond, depicted in Figure 8a, the contributions of the atoms that form the bond account for 96.8% of the electron density at the BCP, whereas 1.5% of the electron density refers to the other H atom in the molecule and 1.7% corresponds to the remaining atoms in the cluster. In a strong HB (see Figure 8b), a similar behavior is exhibited, where the atomic SF contribution of the interacting atoms, $S\%\left(O_{d},H,O_{a}\right)$, is 79.7% and 20.3% is spread along the rest of the water molecules. On the other hand, Figure 8c shows the atomic SF contributions for a weak HB (i.e., $\left|V\left(r_{BCP}\right)\right|/G\left(r_{BCP}\right)<1$), where the SF triad contribution $S\%\left(O_{d},H,O_{a}\right)$ is only 48.1%, and the remaining 51.9% contribution is associated to the remaining atoms in the cluster. The latter observations mean that as the shared-shell character of the HB increases, the SF contributions become more localized and vice versa.

## 3. Models and Methods

The present study considered twelve water clusters ranging from water dimer (H_2_O)_2_ to heptamers (H_2_O)_7_. The initial geometries were obtained from the Refs. [40,46] and further optimized at the M062X/aug-cc-pVTZ level of theory. In this regard, it is important to mention that a recently published benchmark study, focused on the performance of different DFT functionals in the description of hydrogen-bonded systems [68], has determined that the M062X is among the methods providing the lowest RMSE as compared with results obtained by means of highly correlated methods, i.e., CCSD(T). Moreover, this functional has been successfully tested in non-covalent benchmark databases, S66 [69,70]. At this level of calculation, no significant geometric differences were found after the optimization. The error due to the basis set superposition (BSSE) was corrected during the minimization of the energy with respect to the geometry by employing the Boys–Bernardi counterpoise method as implemented in the GAUSSIAN16 suite of programs [71]. Moreover, subsequent vibrational analysis was performed to check the nature of the stationary points. The QTAIM and source function analysis were performed using the AIMALL package [72].

## 4. Conclusions

Non-covalent bonding interactions in twelve different water clusters were investigated in this work. An analysis of the electron density at the BCP between the O and H atoms revealed an inverse relationship between the $\rho \left(r_{BCP}\right)$ value at O_d_–H BCP and its analogous O_a_⋯H, suggesting a charge transfer from the covalent O_d_–H bond to the O_a_⋯H HB. Furthermore, examination of the $\nabla^{2}\rho \left(r_{BCP}\right)$ values evidenced important differences between analogous O_a_⋯H interactions within each cluster. Whereas no noticeable changes were observed in 2D clusters, substantial changes (closed-shell vs. shared-shell character) were detected between different O_a_⋯H interactions in 3D clusters.

SF analysis was also employed to characterize the O_a_⋯H interactions. Unlike 2D clusters, in 3D clusters the S%(H) and $S\%\left(O_{a}\right)$ values varied greatly, which can be explained by the different geometric arrangements that adopt the water molecules. SF analyses also permitted to evaluate the localized/non-localized nature of the O_a_⋯H interactions via $\rho \left(r_{BCP}\right)$ decomposition into atomic contributions. These findings revealed that each individual water molecule affects the entire HB network. Therefore, an accurate description of water cluster interactions beyond the dimer is crucial to obtain accurate potentials for the condensate water simulation.

## Figures and Tables

**Table 1:** Binding energy per water molecule in kcal mol^−1^ for (H_2_O)*_n_* clusters, *n* = 2–7, at M062X/aug-cc-pVTZ + CP and CCSD(T)/CBS/CBS no CP.

Cluster (H_2_O)*_n_*	*n*	Number of HBs	M062X/aug-cc-pVTZ + CP (kcal mol^−1^)	CCSD(T)/CBS/CBS No CP ^1^ (kcal mol^−1^)
2a	2	1	2.57	2.52
3a	3	3	5.74	5.23
4a	4	4	7.12	6.65
4b	4	4	7.38	6.86
5a	5	5	7.68	7.20
6a	6	6	7.90	7.43
6b	6	7	8.16	7.59
6c	6	8	8.45	7.66
6d	6	9	8.56	7.69
7a	7	10	9.02	8.20
7b	7	10	8.99	8.16
7c	7	10	8.99	8.13

^1^ Data from Ref. [46].

**Table 2:** Average source contributions at the BCP of the HB, O⋯H.

Cluster ^1^ (H_2_O)*_n_*	<O⋯O> (Å)	<O⋯H> (Å)	〈|V(r)|/G(r)〉	<S%> (H)	<S%> (O_d_)	<S%> (O_a_)	<S%> (H,O_d_)	<S%> (H,O_a_)	S% (O_d_, H,O_a_)
2a	2.904	1.947	0.966	−37.96	76.10	27.35	38.14	−10.71	65.49
3a	2.783	1.892	0.982	−30.63	67.30	28.93	36.67	−1.70	65.60
4a	2.737	1.774	1.154	−16.79	55.49	35.82	38.70	19.03	74.52
4b	2.728	1.764	1.169	−15.55	54.41	36.18	38.86	20.63	75.04
5a	2.717	1.737	1.206	−13.15	52.47	37.66	39.32	24.51	76.98
6a	2.709	1.734	1.216	−12.37	51.88	38.24	39.51	25.87	77.75

^1^ Only cyclic clusters are considered.

## Data Availability

Not applicable.

## Supplementary Materials

The following supporting information can be downloaded at: https://www.mdpi.com/article/10.3390/ijms24065271/s1.
