# Supplementary material for: Exploring the Non-Covalent Bonding in Water Clusters
Source: Int J Mol Sci. 2023 Mar 9;24(6):5271. doi: 10.3390/ijms24065271 (PMC10049637; doi:10.3390/ijms24065271)
Supplement: Supplementary file 1 [file ijms-24-05271-s001.zip › ijms-2213383-supplementary.pdf]

Supplementary Material

Exploring the non-covalent bonding in water clusters.

Luis E. Seijas[1], Cesar H. Zambrano[2], Rafael Almeida[3], Jorge Alí-Torres[4], Luis Rincón[2] and F. Javier Torres[2,1*]

Figure S1: Water clusters considered, including atom labels (a) $(H_2O)_2$, 2a; (b) $(H_2O)_3$, 3a; (c) $(H_2O)_4$, 4a; (d) $(H_2O)_4$, 4b; (e) $(H_2O)_5$, (f) $(H_2O)_5$, 5a; (f) $(H_2O)_6$, 6a; (g) $(H_2O)_6$, 6b; (h) $(H_2O)_6$, 6c; (i) $(H_2O)_6$, 6d; (j) $(H_2O)_7$, 7a; (k) $(H_2O)_7$, 7b; (l) $(H_2O)_7$, 7c.

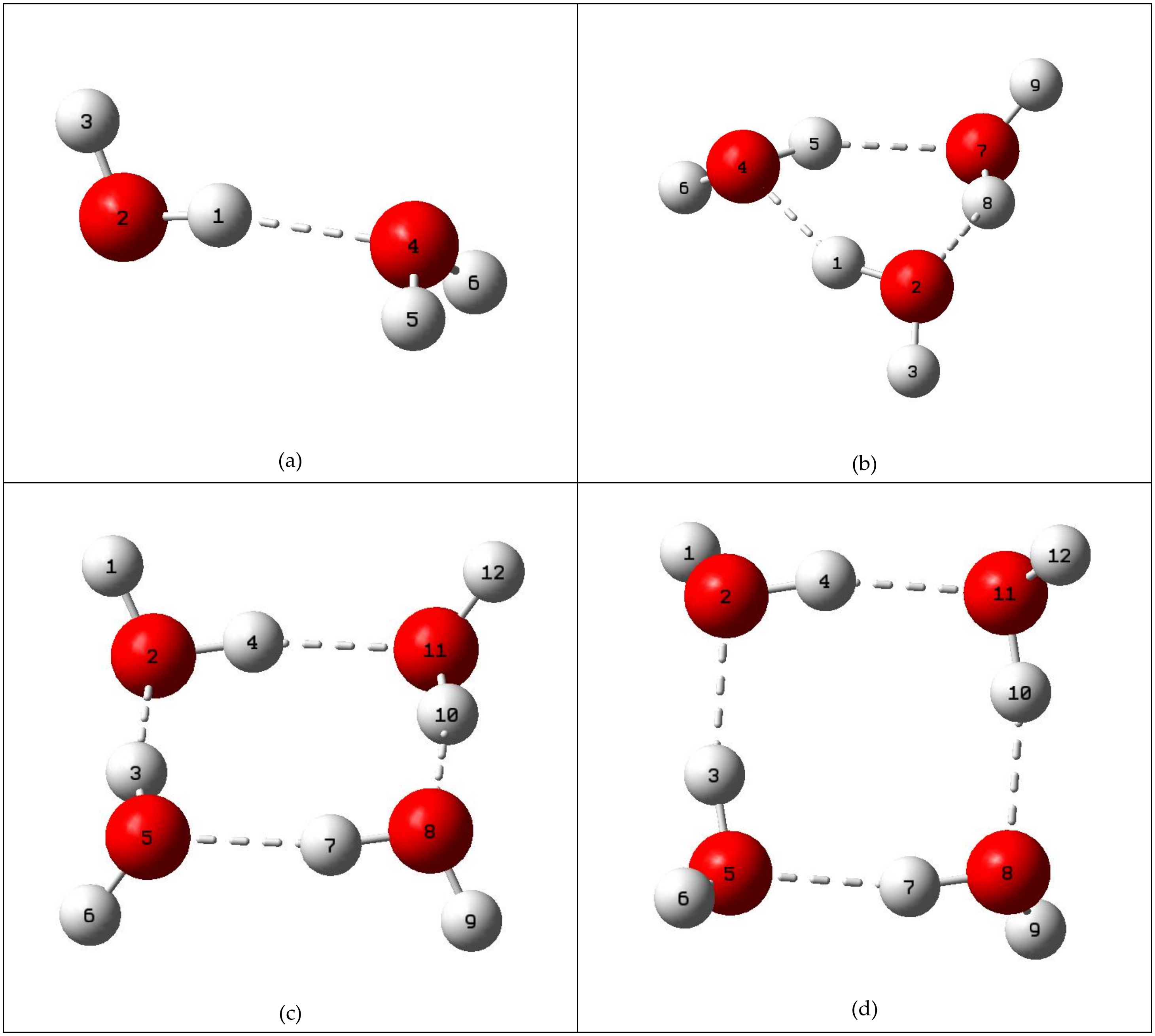

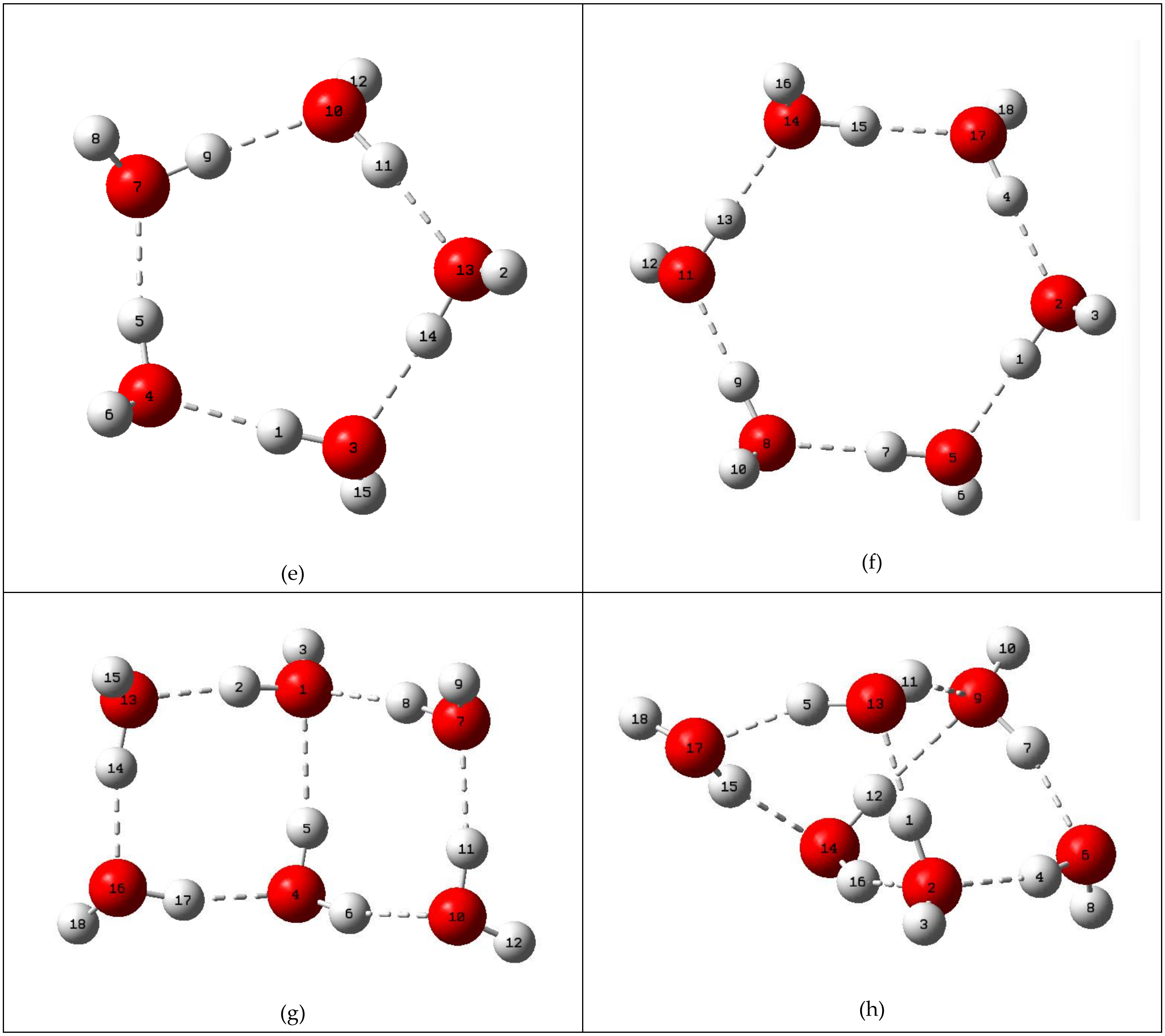
(e)
(f)
(g)
(h)

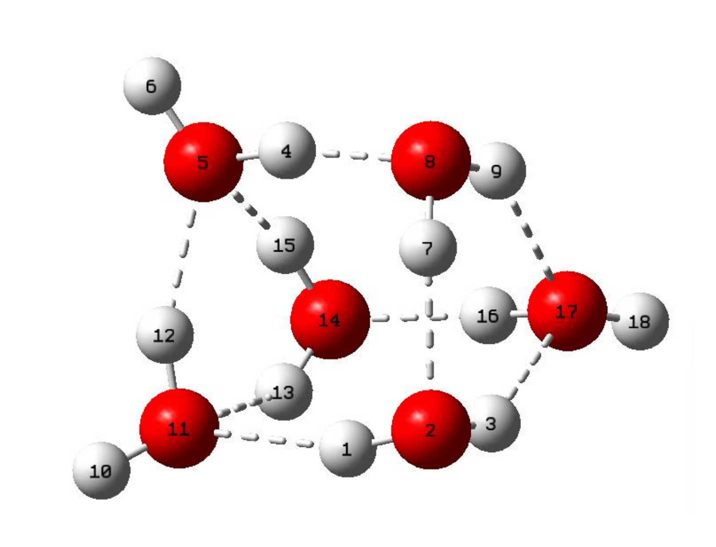
6
5
4
8
9
15
7
14
16
17
18
12
13
11
2
3
1
10
(i)

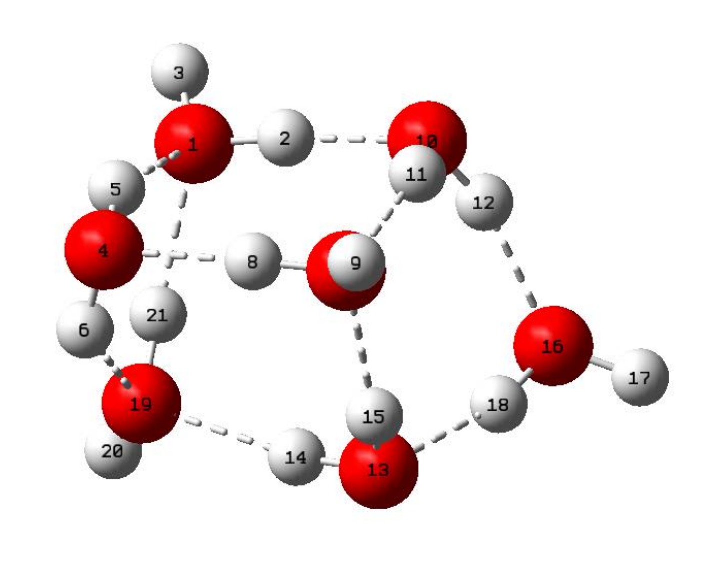
3
1
2
10
11
5
12
4
8
9
6
21
16
17
19
15
18
20
14
13
(j)

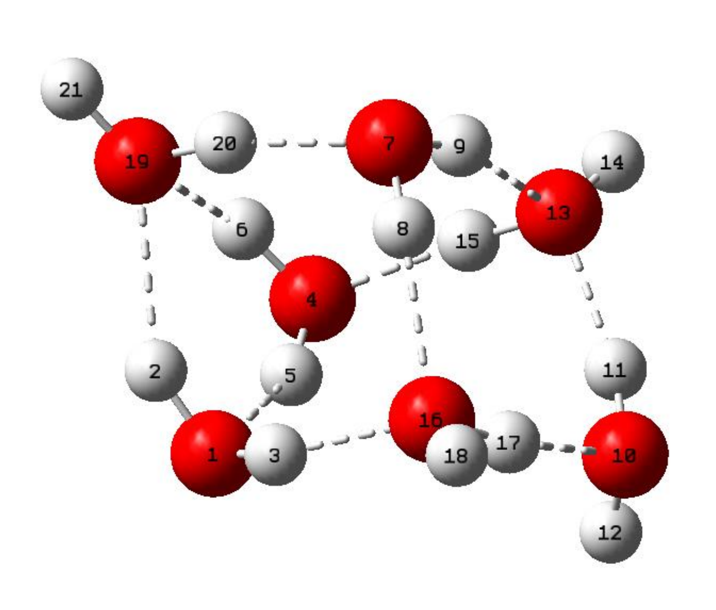
21
19
20
7
9
14
6
13
8
15
4
2
5
11
16
1
3
17
18
10
12
(k)

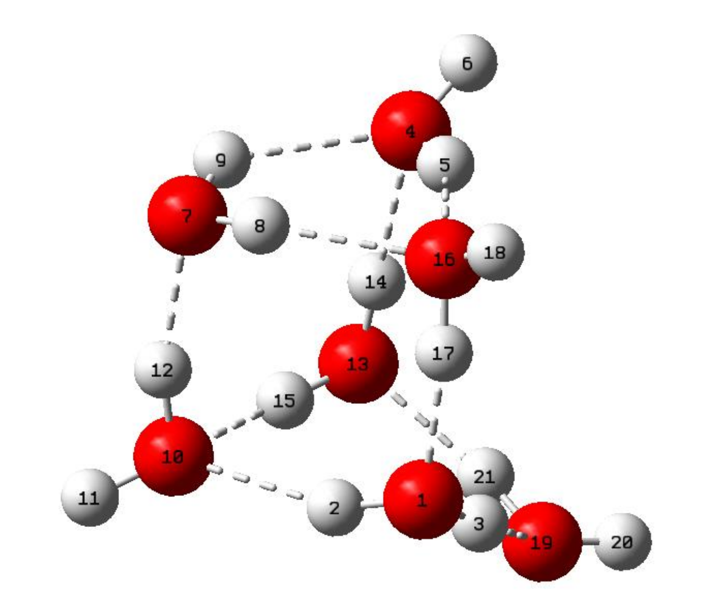
6
4
9
5
7
8
16
18
14
13
17
12
15
10
21
11
2
1
3
19
20
(l)

Table S1. Geometrical parameters and topological properties of HB at $\mathbf{r}_{BCP}$ for each HB found in the considered water clusters.

Model	$O_d—H\cdots O_a$	$H\ldots O_a$ (Å)	$O_d\ldots O_a$ (Å)	$O_d—H\cdots O_a$ (^{0})	$\rho(r_{BCP})$ (e a_0^{-3})	$\nabla^2\rho(r_{BCP})$ (e a_0^{-5})	|V|/G	H (hartree a_0^{-3})
2a	O2-H1...O4	1.947	2.904	170.3	2.41E-02	8.73E-02	0.966	7.13E-04
3a	O2-H1...O4	1.909	2.783	148.8	2.63E-02	9.82E-02	0.966	8.06E-04
3a	O4-H5...O7	1.892	2.785	151.0	2.79E-02	1.00E-01	0.993	1.72E-04
3a	O7-H8...O2	1.874	2.782	150.9	2.76E-02	1.01E-01	0.988	2.95E-04
4a	O2-H4...O11	1.783	2.742	165.8	3.61E-02	1.06E-01	1.138	-4.26E-03
4a	O11-H10...O8	1.765	2.731	168.3	3.82E-02	1.07E-01	1.170	-5.48E-03
4a	O8-H7...O5	1.783	2.742	165.8	3.61E-02	1.06E-01	1.138	-4.25E-03
4a	O5-H3...O2	1.765	2.731	168.2	3.82E-02	1.07E-01	1.170	-5.47E-03
4b	O2-H4...O11	1.764	2.728	167.3	3.83E-02	1.07E-01	1.169	-5.48E-03
4b	O11-H10...O8	1.764	2.728	167.2	3.83E-02	1.07E-01	1.169	-5.48E-03
4b	O8-H7...O5	1.764	2.728	167.3	3.83E-02	1.08E-01	1.169	-5.48E-03
4b	O5-H3...O2	1.764	2.728	167.3	3.83E-02	1.07E-01	1.170	-5.49E-03
5a	O13-H14...O3	1.732	2.711	175.4	4.14E-02	1.08E-01	1.216	-7.45E-03
5a	O10-H11...O13	1.734	2.715	175.0	4.11E-02	1.09E-01	1.211	-7.26E-03
5a	O7-H9...O10	1.735	2.715	176.4	4.12E-02	1.08E-01	1.215	-7.38E-03
5a	O4-H5...O7	1.752	2.728	173.5	3.87E-02	1.08E-01	1.177	-5.82E-03
5a	O3-H1...O4	1.734	2.714	176.2	4.10E-02	1.08E-01	1.211	-7.24E-03
6a	O14-H15...O17	1.734	2.708	178.4	4.16E-02	1.09E-01	1.216	-7.53E-03
6a	O2-H1...O5	1.733	2.708	178.4	4.16E-02	1.10E-01	1.216	-7.53E-03
6a	O17-H4...O2	1.733	2.708	178.4	4.16E-02	1.10E-01	1.216	-7.53E-03
6a	O8--H9…O11	1.735	2.708	178.4	4.16E-02	1.10E-01	1.216	-7.52E-03
6a	O5-H7...O8	1.733	2.708	178.4	4.16E-02	1.09E-01	1.216	-7.54E-03
6a	O11-H13...O14	1.734	2.708	178.4	4.16E-02	1.10E-01	1.216	-7.53E-03
6b	O1-H2...O13	1.685	2.666	171.7	4.71E-02	1.08E-01	1.295	-1.13E-02
6b	O13-H14...O16	1.702	2.675	168.4	4.52E-02	1.09E-01	1.267	-9.92E-03
6b	O16-H17...O4	1.704	2.675	167.0	4.51E-02	1.09E-01	1.264	-9.75E-03
6b	O10-H11...O7	1.808	2.767	166.9	3.38E-02	1.05E-01	1.101	-2.97E-03
6b	O7-H8...O1	1.814	2.779	170.5	3.27E-02	1.05E-01	1.087	-2.48E-03
6b	O4-H6...O10	1.842	2.794	165.2	3.17E-02	1.01E-01	1.072	-1.97E-03
6b	O4-H5...O1	1.978	2.908	160.6	2.27E-02	8.43E-02	0.939	1.22E-03
6c	O6-H4...O2	1.798	2.742	161.5	3.47E-02	1.08E-01	1.106	-3.21E-03
6c	O9-H7...O6	1.754	2.710	162.4	3.96E-02	1.09E-01	1.183	-6.08E-03
6c	O17-H15...O14	1.818	2.755	159.5	3.45E-02	1.03E-01	1.111	-3.24E-03
6c	O14-H16...O2	2.007	2.907	153.8	2.10E-02	8.19E-02	0.902	1.82E-03
6c	O13-H11...O9	1.961	2.869	154.9	2.33E-02	8.85E-02	0.933	1.38E-03
6c	O14-H12...O9	1.976	2.902	159.4	2.33E-02	8.85E-02	0.933	1.38E-03
6c	O2-H1...O13	1.663	2.641	166.7	5.19E-02	1.02E-01	1.368	-1.48E-02
6c	O13-H5...O17	1.851	2.776	157.6	2.99E-02	1.05E-01	1.029	-7.72E-04
6d	O8-H7...O2	1.875	2.778	152.5	3.09E-02	1.01E-01	1.042	-1.11E-03

6d	O5-H4...O8	1.648	2.632	168.9	5.36E-02	1.02E-01	1.387	-1.61E-02
6d	O2-H1...O11	1.962	2.899	162.2	2.33E-02	8.63E-02	0.945	1.12E-03
6d	O11-H12...O5	1.838	2.756	155.7	3.12E-02	1.04E-01	1.050	-1.37E-03
6d	O14-H13...O11	2.077	2.861	137.1	1.82E-02	7.75E-02	0.833	2.78E-03
6d	O14-H15...O5	1.987	2.849	147.2	2.23E-02	8.78E-02	0.905	1.91E-03
6d	O17-H16...O14	1.750	2.715	166.7	4.04E-02	1.07E-01	1.202	-6.77E-03
6d	O2-H3...O17	2.048	2.910	147.4	1.93E-02	7.73E-02	0.867	2.27E-03
6d	O8-H9...O17	2.065	2.868	139.3	1.88E-02	7.80E-02	0.845	2.62E-03
7a	O7-H8...O4	1.722	2.687	166.0	4.39E-02	1.07E-01	1.252	-9.02E-03
7a	O10-H11...O7	1.929	2.854	158.7	2.49E-02	9.21E-02	0.963	8.17E-04
7a	O13-H15...O7	1.930	2.871	162.9	2.49E-02	9.10E-02	0.970	6.57E-04
7a	O10-H12...O16	1.807	2.758	163.6	3.39E-02	1.06E-01	1.100	-2.96E-03
7a	O16-H18...O13	1.753	2.712	164.8	4.03E-02	1.07E-01	1.199	-6.65E-03
7a	O4-H5...O1	1.964	2.829	147.3	2.37E-02	9.16E-02	0.923	1.63E-03
7a	O4-H6...O19	2.089	2.860	135.6	1.79E-02	7.65E-02	0.827	2.82E-03
7a	O13-H14...O19	1.910	2.866	168.1	2.63E-02	9.27E-02	0.996	9.21E-05
7a	O19-H21...O1	1.813	2.742	157.7	3.32E-02	1.07E-01	1.082	-2.39E-03
7a	O1-H2...O10	1.619	2.613	172.2	5.74E-02	1.01E-01	1.426	-1.88E-02
7a	O7-H5...O1	1.964	2.829	147.3	2.37E-02	9.16E-02	0.923	1.63E-03
7b	O4-H5…O1	1.861	2.778	155.1	3.10E-02	1.02E-01	1.052	-1.39E-03
7b	O4-H6...O19	2.030	2.837	139.5	2.00E-02	8.39E-02	0.861	2.56E-03
7b	O19--H20…O7	1.720	2.692	168.2	4.37E-02	1.07E-01	1.251	-8.96E-03
7b	O16--H17…O10	1.723	2.689	166.9	4.27E-02	1.09E-01	1.230	-8.18E-03
7b	O1-H3...O16	1.932	2.870	162.5	2.44E-02	9.08E-02	0.963	8.14E-04
7b	O7-H8...O16	1.906	2.848	162.9	2.65E-02	9.42E-02	0.994	1.35E-04
7b	O13-H15…O4	1.633	2.618	168.8	5.57E-02	1.02E-01	1.409	-1.76E-02
7b	O7-H9...O13	1.949	2.872	158.3	2.39E-02	8.91E-02	0.951	1.04E-03
7b	O10-H11...O13	1.786	2.747	167.0	3.58E-02	1.06E-01	1.134	-4.10E-03
7b	O1-H2...O19	2.036	2.886	145.6	1.98E-02	8.01E-02	0.870	2.30E-03
7c	O7-H9…O4	2.120	2.869	133.2	1.68E-02	7.23E-02	0.811	2.87E-03
7c	O1-H2...O10	1.926	2.851	158.6	2.51E-02	9.23E-02	9.67E-01	7.44E-04
7c	O10-H12…O7	1.728	2.691	164.9	4.30E-02	1.07E-01	1.24E+00	-8.36E-03
7c	O13-H14…O4	1.919	2.883	172.3	2.57E-02	9.07E-02	0.990	2.35E-04
7c	O13-H15…O10	1.945	2.977	160.9	2.41E-02	8.93E-02	0.957	9.18E-04
7c	O4-H5...O16	1.813	2.736	156.5	3.29E-02	1.08E-01	1.074	-2.15E-03
7c	O7-H8...O16	1.949	2.832	150.1	2.46E-02	9.26E-02	0.942	1.26E-03
7c	O16-H17…O1	1.610	2.606	172.2	5.84E-02	1.01E-01	1.436	-1.96E-02
7c	O1-H3...O19	1.813	2.754	161.4	3.23E-02	1.09E-01	1.07	-1.99E-03
7c	O19--H21…O13	1.765	2.716	162.7	3.90E-02	1.07E-01	1.177	-5.78E-03

Table S2. Binding energy per hydrogen bond ($\boldsymbol{BE}[(\mathbf{H_2O})_{\boldsymbol{n}}]/nHB$) in kcal mol^{-1} for $(H_2O)_n$ clusters, n=2–7, at M062X/aug-cc-pVTZ + CP and CCSD(T)/CBS/CBS no CP.

Cluster $(H_2O)_n$	n	Number of HBs	M062X/aug-cc-pVTZ + CP (kcal mol^{-1})	CCSD(T)/CBS/CBS no CP[1] (kcal mol^{-1})
2a	2	1	5.14	5.04
3a	3	3	5.74	5.23
4a	4	4	7.12	6.65
4b	4	4	7.38	6.86
5a	5	5	7.68	7.20
6a	6	6	7.90	7.43
6b	6	7	6.99	6.51
6c	6	8	6.34	5.75
6d	6	9	5.71	5.13
7a	7	10	6.31	5.74
7b	7	10	6.29	5.71
7c	7	10	6.29	5.69

[1] Data from Ref. [47].

Table S3. Electron delocalization Index for each HB found in the considered clusters**.**

Model	O_d—H···O_a	DI(H, O_a)
2a	O2-H1...O4	0.066
3a	O2-H1...O4	0.066
3a	O4-H5...O7	0.070
3a	O7-H8...O2	0.069
4a	O2-H4...O11	0.086
4a	O11-H10...O8	0.090
4a	O8-H7...O5	0.086
4a	O5-H3...O2	0.090
4b	O2-H4...O11	0.090
4b	O11-H10...O8	0.090
4b	O8-H7...O5	0.090
4b	O5-H3...O2	0.090
5a	O13-H14...O3	0.094
5a	O10-H11...O13	0.094
5a	O7-H9...O10	0.094
5a	O4-H5...O7	0.089
5a	O3-H1...O4	0.093
6a	O14-H15...O17	0.094
6a	O2-H1...O5	0.094
6a	O17-H4...O2	0.094
6a	O8--H9…O11	0.094
6a	O5-H7...O8	0.094
6a	O11-H13...O14	0.094
6b	O1-H2...O13	0.103

6b	O13-H14...O16	0.100
6b	O16-H17...O4	0.100
6b	O10-H11...O7	0.082
6b	O7-H8...O1	0.079
6b	O4-H6...O10	0.079
6b	O4-H5...O1	0.063
6c	O6-H4...O2	0.082
6c	O9-H7...O6	0.091
6c	O17-H15...O14	0.084
6c	O14-H16...O2	0.057
6c	O13-H11...O9	0.062
6c	O14-H12...O9	0.061
6c	O2-H1...O13	0.112
6c	O13-H5...O17	0.073
6d	O8-H7...O2	0.077
6d	O5-H4...O8	0.115
6d	O2-H1...O11	0.063
6d	O11-H12...O5	0.076
6d	O14-H13...O11	0.045
6d	O14-H15...O5	0.058
6d	O17-H16...O14	0.093
6d	O2-H3...O17	0.053
6d	O8-H9...O17	0.049
7a	O7-H8...O4	0.099
7a	O10-H11...O7	0.065
7a	O13-H15...O7	0.067
7a	O10-H12...O16	0.082
7a	O16-H18...O13	0.093
7a	O4-H5...O1	0.061
7a	O4-H6...O19	0.044
7a	O13-H14...O19	0.070
7a	O19-H21...O1	0.080
7a	O1-H2...O10	0.119
7a	O7-H5...O1	0.061
7b	O4-H5…O1	0.078
7b	O4-H6--O19	0.051
7b	O19--H20…O7	0.099
7b	O16--H17…O10	0.096
7b	O1-H3...O16	0.065
7b	O7-H8...O16	0.069
7b	O13-H15…O4	0.117
7b	O7-H9...O13	0.064
7b	O10-H11...O13	0.086

7b	O1-H2...O19	0.053
7c	O7-H9…O4	0.041
7c	O1-H2...O10	0.065
7c	O10-H12…O7	0.097
7c	O13-H14…O4	0.069
7c	O13-H15…O10	0.065
7c	O4-H5...O16	0.078
7c	O7-H8...O16	0.063
7c	O16-H17…O1	0.121
7c	O1-H3...O19	0.076
7c	O19--H21…O13	0.091

Table S4. Geometrical parameters and atomic source function contributions to HB $\boldsymbol{\rho}(\mathbf{r}_{BCP})$ for each HB found in the considered water clusters.

Model	O_d—H···O_a	H…O_a (Å)	O_d…O_a (Å)	O_d—H···O_a (⁰)	%S(H)	%S(O_d)	%S(O_a)	%S(H,O_a)	%S(O_d,H,O_a)
2a	O2-H1...O4	1.947	2.904	170.3	-37.96	76.10	27.35	-10.61	65.49
3a	O2-H1...O4	1.909	2.783	148.8	-32.19	68.61	28.26	-3.93	64.68
3a	O4-H5...O7	1.892	2.785	151.0	-29.53	66.53	29.17	-0.36	66.17
3a	O7-H8...O2	1.874	2.782	150.9	-30.16	66.76	29.36	-0.80	65.95
4a	O2-H4...O11	1.783	2.742	165.8	-17.98	56.44	35.48	17.50	73.94
4a	O11-H10...O8	1.765	2.731	168.3	-15.59	54.53	36.16	20.57	75.10
4a	O8-H7...O5	1.783	2.742	165.8	-17.98	56.44	35.49	17.50	73.94
4a	O5-H3...O2	1.765	2.731	168.2	-15.59	54.54	36.15	20.56	75.10
4b	O2-H4...O11	1.764	2.728	167.3	-15.55	54.39	36.21	20.66	75.05
4b	O11-H10...O8	1.764	2.728	167.2	-15.53	54.39	36.18	20.66	75.05
4b	O8-H7...O5	1.764	2.728	167.3	-15.58	54.47	36.13	20.55	75.02
4b	O5-H3...O2	1.764	2.728	167.3	-15.56	54.40	36.21	20.65	75.05
5a	O13-H14...O3	1.732	2.711	175.4	-12.44	51.81	37.90	25.46	77.27
5a	O10-H11...O13	1.734	2.715	175.0	-12.73	52.09	37.80	25.07	77.16
5a	O7-H9...O10	1.735	2.715	176.4	-12.58	52.05	37.74	25.17	77.22
5a	O4-H5...O7	1.752	2.728	173.5	-15.18	54.26	37.04	21.85	76.12
5a	O3-H1...O4	1.734	2.714	176.2	-12.82	52.14	37.81	24.99	77.13
6a	O14-H15...O17	1.734	2.708	178.4	-12.48	51.93	38.24	25.77	77.70
6a	O2-H1...O5	1.733	2.708	178.4	-12.33	51.84	38.27	25.94	77.78
6a	O17-H4...O2	1.733	2.708	178.4	-12.34	51.88	38.21	25.87	77.75
6a	O8--H9…O11	1.735	2.708	178.4	-12.36	51.86	38.26	25.90	77.76
6a	O5-H7...O8	1.733	2.708	178.4	-12.39	51.95	38.15	25.76	77.71
6a	O11-H13...O14	1.734	2.708	178.4	-12.32	51.84	38.28	25.96	77.80
6b	O1-H2...O13	1.685	2.666	171.7	-7.07	46.47	39.24	32.17	78.64
6b	O13-H14...O16	1.702	2.675	168.4	-8.73	48.25	38.56	29.83	78.08
6b	O16-H17...O4	1.704	2.675	167.0	-8.83	48.37	38.67	29.84	78.21

6b	O10-H11...O7	1.808	2.767	166.9	-21.13	59.37	34.42	13.29	72.67
6b	O7-H8...O1	1.814	2.779	170.5	-22.87	60.85	33.11	10.24	71.09
6b	O4-H6...O10	1.842	2.794	165.2	-23.63	61.35	32.62	8.99	70.34
6b	O4-H5...O1	1.978	2.908	160.6	-40.38	74.90	23.22	-17.16	57.74
6c	O6-H4...O2	1.798	2.742	161.5	-19.94	57.81	33.54	13.60	71.41
6c	O9-H7...O6	1.754	2.710	162.4	-14.05	52.51	36.06	22.01	74.52
6c	O17-H15...O14	1.818	2.755	159.5	-19.59	57.97	32.78	13.19	71.17
6c	O14-H16...O2	2.007	2.907	153.8	-44.34	77.44	20.07	-24.27	53.16
6c	O13-H11...O9	1.961	2.869	154.9	-38.73	72.70	24.82	-13.92	58.78
6c	O14-H12...O9	1.976	2.902	159.4	-41.75	75.48	22.80	-18.95	56.53
6c	O2-H1...O13	1.663	2.641	166.7	-3.05	42.61	39.32	36.27	78.89
6c	O13-H5...O17	1.851	2.776	157.6	-26.72	63.05	31.40	4.68	67.73
6d	O8-H7...O2	1.875	2.778	152.5	-23.83	60.05	29.97	6.14	66.19
6d	O5-H4...O8	1.648	2.632	168.9	-2.02	41.83	39.88	37.85	79.69
6d	O2-H1...O11	1.962	2.899	162.2	-39.10	74.19	24.84	-14.26	59.93
6d	O11-H12...O5	1.838	2.756	155.7	-24.55	61.12	31.18	6.63	67.74
6d	O14-H13...O11	2.077	2.861	137.1	-47.16	78.97	16.34	-30.83	48.15
6d	O14-H15...O5	1.987	2.849	147.2	-40.01	73.53	22.30	-17.71	55.82
6d	O17-H16...O14	1.750	2.715	166.7	-13.22	51.95	35.93	22.71	74.66
6d	O2-H3...O17	2.048	2.910	147.4	-47.93	80.76	19.10	-28.82	51.94
6d	O8-H9...O17	2.065	2.868	139.3	-46.65	78.26	18.41	-28.24	50.02
7a	O7-H8...O4	1.722	2.687	166.0	-9.81	48.74	175.79	27.23	214.72
7a	O10-H11...O7	1.929	2.854	158.7	-35.61	70.35	194.34	-9.50	229.08
7a	O13-H15...O7	1.930	2.871	162.9	-35.82	71.10	198.73	-9.83	234.01
7a	O10-H12...O16	1.807	2.758	163.6	-20.95	58.23	184.56	12.91	221.84
7a	O16-H18...O13	1.753	2.712	164.8	-13.32	52.48	178.11	23.10	217.27
7a	O4-H5...O1	1.964	2.829	147.3	-36.95	70.92	184.20	-12.94	218.18
7a	O4-H6...O19	2.089	2.860	135.6	-47.12	78.63	182.73	-31.64	214.24
7a	O13-H14...O19	1.910	2.866	168.1	-32.94	68.97	201.07	-5.02	237.10
7a	O19-H21...O1	1.813	2.742	157.7	-21.81	58.82	179.55	10.68	216.57
7a	O1-H2...O10	1.619	2.613	172.2	0.27	39.77	171.88	41.65	211.92
7a	O7-H5...O1	1.964	2.829	147.3	-36.95	70.92	184.20	-12.94	218.18
7b	O4-H5…O1	1.861	2.778	155.1	-23.76	60.30	30.44	66.98	66.98
7b	O4-H6--O19	2.030	2.837	139.5	-43.92	76.38	19.81	52.27	52.27
7b	O19--H20…O7	1.720	2.692	168.2	-10.05	49.11	37.54	76.60	76.60
7b	O16--H17…O10	1.723	2.689	166.9	-11.15	49.95	37.78	76.58	76.58
7b	O1-H3...O16	1.932	2.870	162.5	-37.03	72.78	25.49	61.24	61.24
7b	O7-H8...O16	1.906	2.848	162.9	-32.39	68.17	27.62	63.40	63.40
7b	O13-H15…O4	1.633	2.618	168.8	-0.80	40.63	40.59	80.42	80.42
7b	O7-H9...O13	1.949	2.872	158.3	-37.76	72.34	24.41	58.98	58.98
7b	O10-H11...O13	1.786	2.747	167.0	-18.60	56.74	34.05	72.20	72.20
7b	O1-H2...O19	2.036	2.886	145.6	-46.37	79.19	19.76	52.57	52.57
7c	O7-H9…O4	2.120	2.869	133.2	-48.82	79.57	13.55	44.30	44.30

7c	O1-H2...O10	1.926	2.851	158.6	-35.31	70.03	26.43	61.15	61.15
7c	O10-H12…O7	1.728	2.691	164.9	-10.72	49.52	36.83	75.63	75.63
7c	O13-H14…O4	1.919	2.883	172.3	-34.34	70.20	27.50	63.36	63.36
7c	O13-H15…O10	1.945	2.977	160.9	-37.25	72.43	25.20	60.38	60.38
7c	O4-H5...O16	1.813	2.736	156.5	-22.29	59.40	32.36	69.47	69.47
7c	O7-H8...O16	1.949	2.832	150.1	-35.30	69.77	24.87	59.33	59.33
7c	O16-H17…O1	1.610	2.606	172.2	0.84	39.28	41.72	81.84	81.84
7c	O1-H3...O19	1.813	2.754	161.4	-23.35	60.42	33.43	70.49	70.49
7c	O19--H21…O13	1.765	2.716	162.7	-14.71	53.84	35.90	75.03	75.03
